# Is it now time to iron out the wrinkles? Health of Shar Pei dogs under primary veterinary care in the UK

**DOI:** 10.1186/s40575-023-00134-z

**Published:** 2023-12-14

**Authors:** Dan G. O’Neill, Karolina S. Engdahl, Alice Leach, Rowena M. A. Packer, David B. Church, Dave C. Brodbelt

**Affiliations:** 1 Pathobiology and Population Sciences, The Royal Veterinary College, Hawkshead Lane, North Mymms, Hatfield, Herts AL9 7TA UK; 2 Department of Clinical Sciences, Swedish University of Agricultural Sciences, PO Box 7054, 750 07 Uppsala, Sweden; 3 Clinical Science and Services, The Royal Veterinary College, Hawkshead Lane, North Mymms, Hatfield, Herts AL9 7TA UK

**Keywords:** VetCompass, Electronic patient record, EPR, Breed, Dog, Epidemiology, Primary-care, Veterinary, Pedigree, Purebred

## Abstract

**Background:**

The Shar Pei is a common dog breed with a distinctive appearance caused by hyaluronosis that has been linked with several health conditions. Anonymised primary-care veterinary clinical records were explored to extract data on the demography, common disorders and mortality of Shar Pei in the UK in 2013.

**Results:**

The study population of 455,557 dogs included 1913 (0.42%) Shar Pei. The mean adult bodyweight was 22.26 kg. The most prevalent fine-level precision disorders were entropion (prevalence 17.88%, 95% CI: 16.16-19.59), otitis externa (16.36%, 95% CI: 14.70-18.02), ear disorders (6.69%, 95% CI: 5.57-7.81), aggression (5.23%, 95% CI: 4.23-6.22), and pyoderma (4.29%, 95% CI: 3.38-5.19). The most prevalent disorder groups were ophthalmologic (prevalence = 22.27%, 95% CI: 20.40-24.13), dermatological (21.01%, 95% CI: 19.19-22.84), aural (18.66%, 95% CI: 16.92-20.41), traumatic injury (7.53%, 95% CI: 6.35-8.71) and behavioural (7.21%, 95% CI: 6.05-8.37). The median longevity of 190 Shar Pei that died during the study period was 7.28 years (IQR 5.04-10.05, range 0.04-15.04). Of 184 deaths with a recorded method of death, 157 (85.33%) deaths involved euthanasia and 27 (14.67%) deaths were unassisted. Among 136 (71.58%) deaths with a recorded biomedical cause of death, the most common causes of death at group level precision were neoplasia (15.44%, 95% CI: 9.37-21.51), renal disorders (13.24%, 95% CI: 7.54-18.93), and behavioural disorders (11.03%, 95% CI: 5.76-16.29).

**Conclusions:**

Almost one fifth of Shar Pei receive veterinary care each year for entropion, a condition linked strongly with the extreme conformation of thickened and folded skin and bristly hair that characterises the Shar Pei breed. Several other common disorders are also linked to hyaluronosis. Current UK legislation can help support efforts to avoid breeding or acquiring animals with extreme conformations and to promote adequate veterinary care for already-owned animals with extreme conformations.

## Abstract (plain language summary)

The Shar Pei dog breed is instantly recognisable by their copious loose folded skin and bristly coat, with Shar Pei translating from Chinese as ‘sand paper skin’. Although originally a fighting, hunting and guarding dog in China, the Shar Pei breed was re-invented in the US as a companion animal in the late 1970s where a more wrinkled and heavy-set American ‘meat mouth’ variety replaced the traditional ‘bone mouth’ variety originally popular in China. However, the underlying disease of hyaluronosis that causes the characteristic extreme appearance of the Shar Pei has also been linked with several other health problems in the breed such as in-turned eyelids (entropion) and ear disease. Using anonymised veterinary clinical records collected within the VetCompass Programme at the Royal Veterinary College (RVC), Shar Pei made up 0.4% of all dogs in the UK in 2013 and had an average adult bodyweight of 22 kg. The most common specific disorders diagnosed in Shar Pei were in-turned eyelids (17.88%), ear infection (16.36%), ear problems (6.69%), aggression (5.23%), and skin infection (4.29%). Among the 30 most common specific disorders, females were more likely to show in-turned eyelids and conjunctivitis, while males were more likely to show aggression and infected paws. Once the disorders were grouped, the most common disorder groups were eye disorders (22.27%), skin (21.01%), ear (18.66%), traumatic injury (7.53%), and behavioural (7.21%). The average lifespan of Shar Pei was 7.28 years. The most common causes of death were cancer (15.44%,), kidney disorders (13.24%), and behavioural disorders (11.03%). This study highlights that almost one fifth of the breed receive veterinary care each year for in-turned eyelids that can lead to extreme pain and are linked to the thickened and folded skin that humans find so appealing in this breed. Current UK legislation can help support concerted efforts from all animal welfare stakeholders to avoid breeding or acquiring animals with these and other extreme conformations and to promote adequate veterinary care for already-owned animals with extreme conformations.

## Background

The Shar Pei breed describes a highly recognisable medium-sized type of dog grouped by The Kennel Club (KC) as a utility breed [1]. Originally invented in China as a fighting, hunting and guarding dog [2], dogs of the modern breed type are instantly recognisable and defined by their copious loose thickened and folded skin and bristly coat, with Shar Pei translating from Chinese as ‘sand paper skin’ [1]. Their unique skin and coat were originally purported to enhance their fighting abilities by making it difficult for opponents to grab and hold onto them while their pliable loose skin allowed the Shar Pei to turn on their opponents even if grabbed in a fight [3]. From near extinction following heavy taxation of dog ownership under the Mao Zedong regime in China, the Shar Pei breed concept was resurrected as a companion animal after a small population of Shar Pei were exported to America as part of a “Save the Shar Pei” movement in the late 1970s [4]. This developed a new US market for Shar Pei as a commodity with a public demand for a much more extreme phenotype than the original type of dog in China, leading to the more wrinkled and heavy-set American ‘meat mouth’ variety of Shar Pei being much more common today than the traditional ‘bone mouth’ variety originally popular in China [3].

Unfortunately, however, the extreme dermatological characteristics that make the modern Shar Pei uniquely recognisable also promote several serious health concerns in the breed [5]. The Shar Pei is reported with predisposition to 29 distinct disorders [6]. The UK Kennel Club’s ‘Breed Watch’ system has identified points of concern for special attention by judges to include excessive amounts of loose facial skin with conformational defects of the upper and/or lower eyelids so that the eyelid margins are not in normal contact with the eye, hair loss or scarring from previous dermatitis, lower lip folding over lower incisors and signs of dermatitis in skin folds [7]. Annual proportional registration with the Kennel Club has dropped from 0.81% of all registrations in 2013 to 0.30% in 2021 [8]. However, the Shar Pei is still a relatively common breed and was the 43rd most frequently owned breed from 800 breeds under veterinary care in the in the UK in 2019, representing 0.30% of all dogs [9]. Any reducing public demand to own these dogs may reflect growing awareness of the high prevalence of health issues in the breed. Therefore strengthening the evidence base on common disorders and mortality of the breed could support welfare-focused reform to improve the quality of life for future dogs by promoting moves away from extremes of conformation. This could also protect the Shar Pei breed concept itself from extinction following growing legal and ethical efforts to promote better innate health in those dogs we choose to breed and own [10, 11].

In the Shar Pei breed, a genetic mutation in the hyaluronic acid synthase 2 (HAS2) gene causes the hyaluronosis disease that gives these dogs their distinctive folded and thickened skin. Over-expression of HAS2 in dermal fibroblasts results in deposition of excess hyaluronic acid throughout the skin, leading to especially thickened skin folds around the head and tibiotarsal joints [12, 13]. Although almost every Shar Pei is affected by hyaluronosis to some degree, the extent of the abnormalities varies among individuals and ages, with puppies also showing greater skin folding than adult dogs. However, increasing modern public demand for the more extreme phenotypes has meant that producers have selected for the more heavily wrinkled ‘meatmouth’ Shar Pei variant to meet demand for dogs that retain their skin folds into adulthood [13, 14]. In addition to issues related to the skin folds, hyaluronic acid is also the main mucin component in cutaneous mucinosis, a skin disease which is commonly reported in Shar Pei [12, 15]. However, good generalisable evidence on the extent to which these hereditary conditions contribute to the overall disorder burden of Shar Pei dogs in the general population has been lacking but would contribute to evidence-based reforms to the breed phenotype that could protect the welfare of future generations of these dogs.

Extreme conformation in dogs is defined as a ‘physical appearance that has been so significantly altered by humankind away from the ancestral natural canine appearance that affected dogs commonly suffer from poor health and welfare, with negative impacts on their quality or quantity of life ‘ [11]. Skin folding meets these criteria for an extreme conformation because of its strong breed predisposition reflecting human selective pressure and links to severe disorders such as skin fold dermatitis and entropion that often result in lifelong reduced quality of life [16–18]. Analysis of primary care veterinary clinical records in the UK reported a 0.91% annual prevalence of skin fold dermatitis diagnosis in the Shar Pei that represented a 6.4 times higher odds in the Shar Pei compared to crossbred dogs [17]. Thickened and folded skin in the ear canals combined with stenotic and hypoplastic ear canals have also been reported to predispose the Shar Pei to otitis externa [19]. In the UK, the Shar Pei has been reported with 3.4 times the odds of otitis externa compared to crossbreed dogs [20]. An increasing evidence base on predisposition to those health conditions linked with extreme conformations in dogs is leading to growing momentum to give greater priority to canine health over the aesthetic wishes of humans for the types of dogs we own, and is supporting wider collaborative efforts to remove the ‘social license to operate’ that has previously permitted celebration of extreme conformations [11, 21].

There is substantial evidence supporting disorders of the eyes and their adnexa as being common and severe in the Shar Pei [6]. As well as inherited ocular issues such as hereditary glaucoma [22] and hereditary primary lens luxation [23], some small-scale studies have highlighted entropion as a particularly severe and common disorder in the Shar Pei that is compounded by selection for facial conformation with heavy skin folds to meet public demand [24]. That ‘Blue Book’ of ocular disorders presumed to be inherited in purebred dogs also flags common occurrence in the Shar Pei of chronic keratitis that is secondary to either entropion or a combination of entropion and ectropion. However it is also recognised that the quality and generalisability of evidence for entropion in the Shar Pei is weak, with the ‘Blue Book’ relying on entropion prevalence data generated from a relatively small sample showing 27/94 (28.7%) Shar Pei assessed in the US had entropion [24]. Information on the fuller scale of ocular and adnexal disorders in the Shar Pei based on larger samples from the general population of dogs could support wider efforts by many stakeholders to move the dog-owning public away from cherishing extreme conformations in the dogs they acquire [11, 21, 25].

In addition to predisposing to skin disorders, increased serum concentration of hyaluronic acid in the Shar Pei breed is considered to also underlie predisposition to high levels of autoinflammatory disorders in the breed [13]. Familial Shar Pei fever is one of these predisposed autoinflammatory disorders, with affected individuals showing recurrent episodes of pyrexia without a clear inciting cause, and generally accompanied by swelling of the joints and muzzle [26]. A study of Shar Pei dogs owned in the UK reported that 49% (52/106) had at least one episode of fever attributed by a veterinarian to Shar Pei autoinflammatory disease [5]. Shar Pei are additionally reported with predispositions for other inflammatory conditions including skin disease, otitis externa, inflammatory chronic enteropathies and amyloidosis that all fall within an overall syndrome called Shar Pei autoinflammatory disease (SPAID) [27, 28]. Accumulation of hyaluronic acid has been shown to trigger a reactive amyloidosis with amyloid deposits in the kidney, liver, spleen, gastrointestinal tract and other locations in the Shar Pei [29]. Renal amyloidosis as a progressive and fatal condition is reported to occur at a younger age in Shar Pei than other dog breeds [30]. However, there is limited evidence on the prevalence of diagnosis of Shar Pei fever, amyloidosis and other autoinflammatory disorders in the wider population of Shar Pei in the UK that would be needed to fully understand the overall welfare cost at a breed level [31].

Using anonymised veterinary clinical data from the VetCompass™ Programme [32], this study aimed to characterise the demography, common disorders and longevity of the general population of Shar Pei dogs under primary veterinary care in the UK. The study placed special focus on comparing results between males and females. Based on both the direct outputs of the current study and following comparison to results for other breeds based on VetCompass studies using similar methods, these results could support initiatives for improved breeding, acquiring and clinical management that ultimately contribute to better health and welfare of Shar Pei dogs [11, 25].

## Materials and methods

The study population included all dogs under primary veterinary care at clinics participating in the VetCompass™ Programme during 2013 [32]. Dogs under veterinary care were defined as those with either a) at least one electronic patient record [EPR] (free-text clinical note, treatment, or bodyweight) recorded during 2013 or b) at least one EPR recorded both before and after 2013 during the available clinical records. VetCompass collates de-identified EPR data from primary-care veterinary practices in the UK for epidemiological research. VetCompass data fields available for the current study included fixed variables of unique animal identifier, species, breed, date of birth, sex and neuter status along with time-varying variables of bodyweight, free-form text clinical notes and treatment with relevant dates.

Dogs recorded as Shar Pei were categorised as Shar Pei while all remaining dogs were categorised as non-Shar Pei. *All-age Bodyweight* (Kg) described all available bodyweight and date combinations for each dog. *Adult Bodyweight* (Kg) described the mean of all bodyweight values for dogs aged ≥ 18 months and was categorised into 7 groups (< 18.0, 18.0 to < 20.0, 20.0 to < 22.0, 22.0 to < 24.0, 24.0 to < 26.0, 26.0 to < 28, ≥ 28.0). *Neuter* (entire or neutered) described the status of the dog at the final EPR. *Age* (years) described the age at the final date under veterinary care during 2013 (December 31st, 2013) and was categorised into 8 groups (< 1.0, 1.0 to < 2.0, 2.0 to < 3.0, 3.0 to < 5.0, 5.0 to < 7.0, 7.0 to < 9.0, 9.0 to < 11.0, ≥ 11.0).

A cohort study design was used to estimate the 1-year period prevalence of the most diagnosed disorders of Shar Pei dogs from a population of 455,557 dogs across all breeds under primary veterinary care during 2013 at VetCompass participating practices.

Given the interest in the current study to compare disorder risk between the sexes, sample size calculation using OpenEpi software (http://www.openepi.com) estimated that a sample of 1774 dogs were needed to detect a risk ratio of 1.5 or greater for a disorder that occurred in 5% of dogs with power of 80% and 95% significance, assuming equal numbers of males and female. Ethics approval was obtained from the RVC Ethics and Welfare Committee (reference number 2015/1369).

The EPRs of all available Shar Pei were manually reviewed in detail to extract the most definitive diagnoses recorded for all disorders recorded as existing during 2013 and to link these to the most appropriate VeNom term as previously described [33]. The extracted diagnosis terms were mapped to a dual hierarchy of precision for analysis: fine-level precision and grouped-level precision [33]. Fine-level precision terms described the original extracted terms at the maximal diagnostic precision recorded within the clinical notes (e.g. *inflammatory bowel disease* remained as *inflammatory bowel disease*). Grouped-level precision terms mapped the original diagnosis terms to a general level of diagnostic precision (e.g. *inflammatory bowel disease* mapped to *enteropathy)*. Disorders described within the clinical notes using presenting sign terms (e.g. ‘vomiting’ or ‘vomiting and diarrhoea’) without a formal clinical diagnostic term were included using the first sign listed (e.g. vomiting). Elective (e.g. neutering) or prophylactic (e.g. vaccination) clinical events were excluded. No distinction was made between pre-existing and incident disorder presentations. The epidemiological unit for the current study was each individual Shar Pei dog for the single year of the study (2013). No information was extracted on the owner’s original rationale for seeking veterinary care (e.g. seeking prophylactic care versus presenting a dog with prior awareness of health issues) across the range of veterinary interactions that may have occurred during this year of interest. Mortality data (recorded cause, date and method of death) were extracted on all deaths at any date during the available EPRs.

Following data checking for internal validity and cleaning in Excel (Microsoft Office Excel 2013, Microsoft Corp.), analyses were conducted using R version 4.0.0 [34]. Annual proportional birth rates described the relative proportion of Shar Pei compared with all dogs born in each year from 2003 to 2013 from the cohort under veterinary care in 2013. The figure illustrating annual proportional birth rates was generated with the R package ggplot2 [35]. All bodyweight data with their associated dates at any age were used to generate individual bodyweight growth curves for male and female Shar Pei by plotting age-specific bodyweights overlaid with a cross medians line using the R package ggplot2 [35].

One-year (2013) period prevalence was reported along with 95% confidence intervals (CI) that described the probability of diagnosis at least once during 2013. The CI estimates were derived from standard errors based on approximation to the normal distribution (Wald CI) for disorders with ten or more events or the Wilson approximation method for disorders with fewer than ten events, using the binom.approx() and binom.wilson() functions from the R package epitools [36]. Prevalence values were reported overall and separately for males and females. Median age (years) was reported for each of the most common diagnoses at fine-level and group-level. The 10 most common disorders at group-level precision in each of three age bands (< 2 years, 2-5 years, and > 5 years) were identified and the prevalence of each these disorders through life up to the age of 11 years was presented using loess curves in a figure generated with the R packages ggplot2, cowplot, and ggpubr [35, 37, 38]. A combination of the Shapiro-Wilk test and visual assessment of histograms was used to assess normality of continuous variables. The two-proportion *z*-test was used to compare proportions, the chi-square test to compare categorical variables, and the Mann-Whitney *U* test and Student’s *t*-test to compare non-normally and normally distributed continuous variables, respectively [39]. Statistical significance was set at the 5% level.

## Results

### Demography

**Fig. 1:**
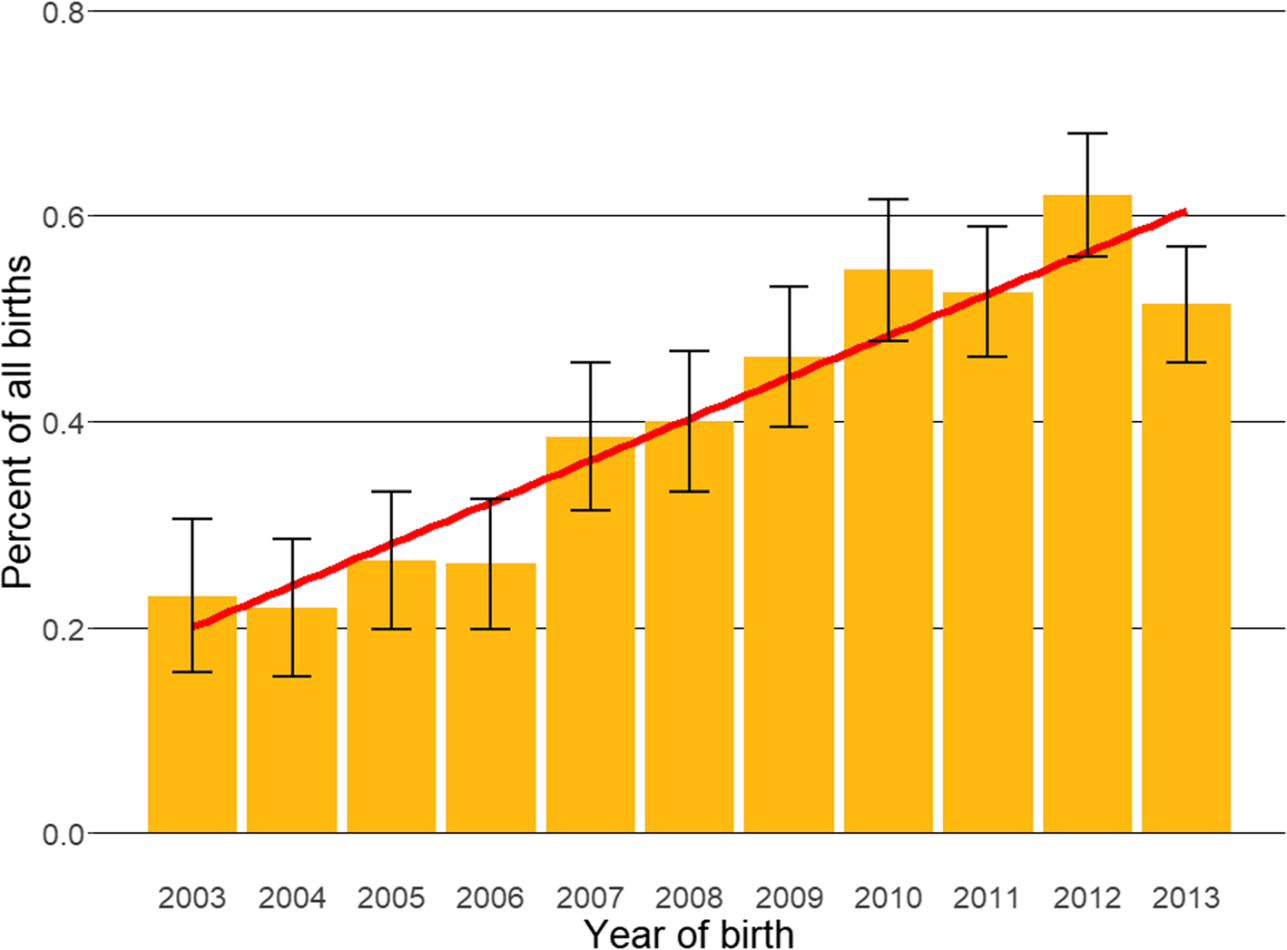
Annual proportional birth rates (2003-2013) with linear trend and 95% confidence intervals for Shar Pei (*n = 1892)* among all dogs with information on birth year (*n = 447,541)* under UK primary veterinary care from January 1st to December 31st, 2013 at practices participating in the VetCompass™ Programme

The study population of 455,557 dogs under veterinary care during 2013 included 1913 (0.42%) Shar Pei. The study included 304 clinics drawn from three larger veterinary groups as well as 3 individual clinics participating in VetCompass. The locations of these clinics were distributed across all areas of the UK. Of the Shar Pei with information available, 931 (48.74%) were female and 572 (32.93%) were neutered (Table 1). Proportional neuter status did not differ statistically significantly between females (30.90%) and males (34.99%) (chi-square test: *P* = 0.078). The overall median age of Shar Pei in the study was 2.74 years (interquartile range (IQR) 1.22-5.15, range 0.05-15.78). Annual proportional birth rates showed increasing breed popularity during 2003-2013, from 0.23% of births of all dogs during 2003 to 0.51% of births 2012 (Fig. 1).

**Table 1:** Demography of Shar Pei dogs under primary veterinary care at practices participating in the VetCompass™ Programme in the UK from January 1st to December 31st, 2013. *n* = 1913. ^a^Counts cover dogs with available data

Variable	Category	Female no. (%)^a^	Male no. (%)^a^	Total no. (%)^a^
Neutered		262 (30.90)	310 (34.99)	572 (32.93)
Bodyweight (kg)	< 18	111 (22.11)	32 (5.94)	143 (13.74)
	18 to < 20	93 (18.53)	49 (9.09)	142 (13.64)
	20 to < 22	102 (20.32)	102 (18.92)	204 (19.60)
	22 to < 24	81 (16.14)	115 (21.34)	196 (18.83)
	24 to < 26	59 (11.75)	84 (15.58)	143 (13.74)
	26 to < 28	26 (5.18)	67 (12.43)	93 (8.93)
	> 28	30 (5.98)	90 (16.70)	120 (11.53)
Age (years)	< 1.0	152 (16.58)	163 (16.77)	316 (16.70)
	1.0 to < 2.0	207 (22.57)	194 (19.96)	402 (21.25)
	2.0 to < 3.0	114 (12.43)	152 (15.64)	266 (14.06)
	3.0 to < 5.0	199 (21.70)	215 (22.12)	415 (21.93)
	5.0 to < 7.0	129 (14.07)	115 (11.83)	244 (12.90)
	7.0 to < 9.0	53 (5.78)	73 (7.51)	126 (6.66)
	9.0 to < 11.0	39 (4.25)	39 (4.01)	78 (4.12)
	≥ 11.0	24 (2.62)	21 (2.16)	45 (2.38)

**Fig. 2:**
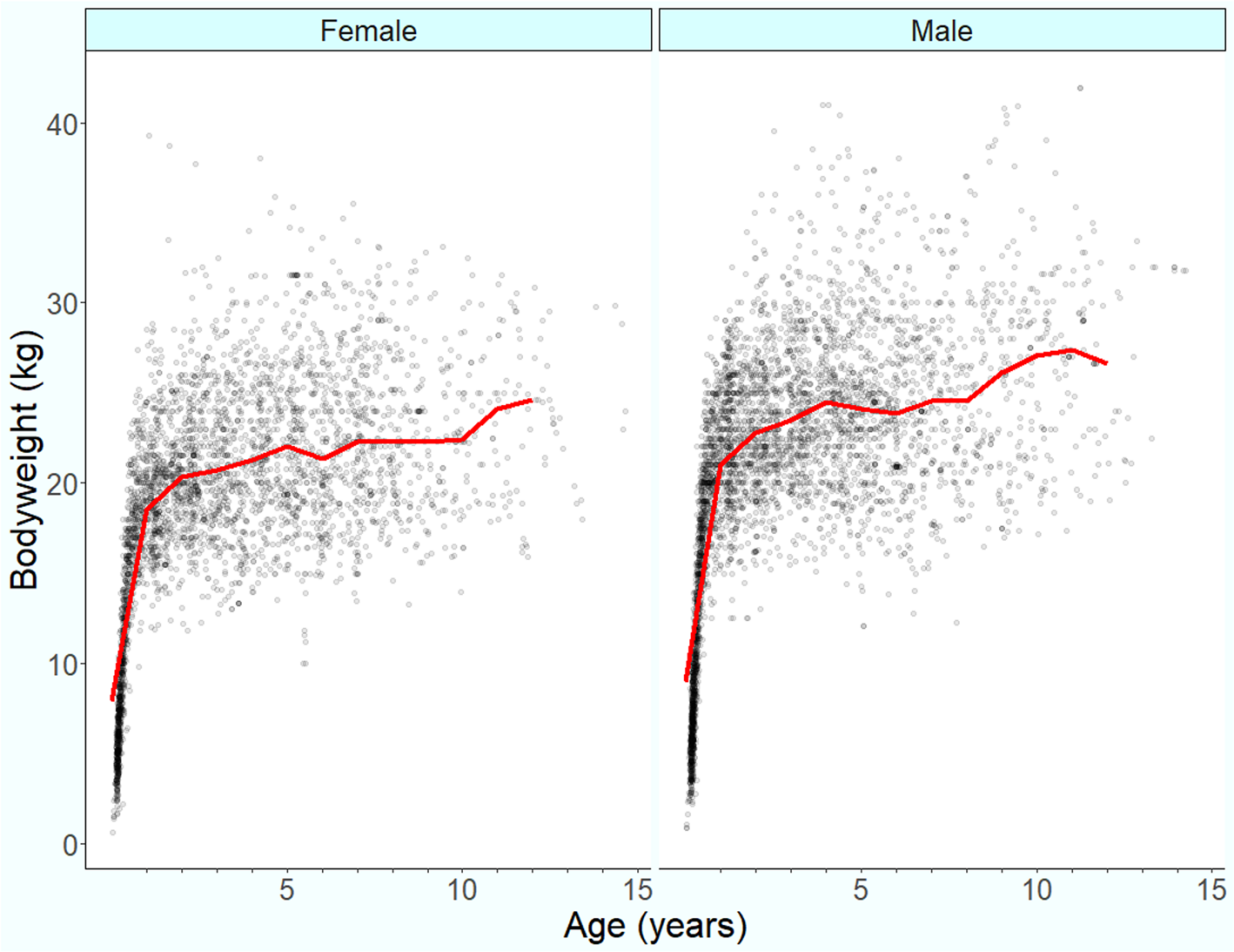
Bodyweight at different ages with a cross medians line plot for female (*n =* 713*)* and male (*n* = 759*)* Shar Pei dogs under UK primary veterinary care from January 1st to December 31st, 2013 at practices participating in the VetCompass™ Programme

The median adult bodyweight of Shar Pei was 22.26 kg (IQR 19.67-25.21, range 10.00-39.50). Males (23.50 kg, IQR 20.93-26.70, range 13.00-39.50) were heavier than females (20.76 kg, IQR 18.55-23.65, range 10.00-35.75) (Mann-Whitney *U* test: *P* = 0.001). Median bodyweight across all ages was higher in males (19.88, IQR 14.44-23.35, range 0.89-39.50) than in females (17.39, IQR 12.60-21.10, range 0.65-35.00) (Mann-Whitney *U* test: *P* < 0.001). Bodyweight curves based on 4388 bodyweight values in 759 males and 3717 bodyweight values in 713 females showed that Shar Pei dogs grow rapidly during their first year and continue to gain weight until around four (males) to five (females) years of age (Fig. 2). Proportional completeness for each variable was sex 99.84%, neuter 90.80%, mean adult bodyweight 54.42% and age 98.90%.

### Disorder prevalence

Overall, 1268 (66.28%) Shar Pei had at least one disorder recorded during 2013. There were 2778 unique disorder events reported across the 1913 Shar Pei during 2013. The median annual disorder count per Shar Pei was 1 (IQR 0-2, range 0-9). The medial annual disorder count did not differ significantly between females (1, IQR 0-2, range 0-9) and males (1, IQR 0-2, range 0-9) (Fisher’s exact test, *P* = 0.176).

Of the 277 fine-level disorder terms reported during 2013, the most common were entropion (*n =* 342, prevalence 17.88%, 95% CI: 16.16-19.59), otitis externa (*n =* 313, 16.36%, 95% CI: 14.70-18.02), ear disorders (*n =* 128, 6.69%, 95% CI: 5.57-7.81), aggression (*n =* 100, 5.23%, 95% CI: 4.23-6.22), and pyoderma (*n =* 82, 4.29%, 95% CI: 3.38-5.19) (Table 2). Specifically, Shar Pei fever was the ninth most commonly diagnosed disorder, with a prevalence of 3.03% (*n* = 58, 95% CI 2.26-3.80). Among the 30 most common fine-level disorders, females had higher probability of entropion and conjunctivitis, while males had higher probability of aggression and pododermatitis (two-proportion *z*-test: *P* ≤ 0.05). The median age of dogs recorded with the 30 most common fine-level diagnoses varied from 1.02 years for umbilical hernia to 6.81 years for periodontal disease (Table 2).

**Table 2:** Prevalence of the most common disorders at fine-level diagnostic precision in Shar Pei dogs (*n* = 1913) under primary veterinary care at practices participating in the VetCompass™ Programme in the UK from January 1st to December 31st, 2013. * *P*-value for prevalence comparison between male and female with two-proportion *z*-test

Fine-level disorder	Count	Prevalence (%)	Female prevalence (%)	Male prevalence (%)	*P*-value*	Median age (years) of dogs with the condition
Entropion	342	17.88 (16.16 - 19.59)	19.76	16.04	0.039	1.38
Otitis externa	313	16.36 (14.70 - 18.02)	17.40	15.42	0.269	2.68
Ear disorder	128	6.69 (5.57 - 7.81)	5.80	7.46	0.174	3.11
Aggression	100	5.23 (4.23 - 6.22)	2.36	7.97	< 0.001	3.36
Pyoderma	82	4.29 (3.38 - 5.19)	4.73	3.88	0.425	2.16
Conjunctivitis	77	4.03 (3.14 - 4.91)	5.16	2.86	0.014	1.60
Dog bite injury	76	3.97 (3.10 - 4.85)	3.97	3.98	0.999	2.86
Cutaneous disorder	59	3.08 (2.31 - 3.86)	3.11	3.06	0.999	3.28
Shar Pei fever	58	3.03 (2.26 - 3.80)	2.90	3.17	0.837	4.45
Atopic dermatitis	57	2.98 (2.22 - 3.74)	3.44	2.55	0.317	4.65
Ulcerative keratitis	52	2.72 (1.99 - 3.45)	2.69	2.76	0.999	1.61
Pruritus	47	2.46 (1.76 - 3.15)	1.93	2.96	0.193	3.00
Skin mass	38	1.99 (1.36 - 2.61)	2.15	1.84	0.749	4.55
Corneal disorder	36	1.88 (1.27 - 2.49)	1.83	1.94	0.987	1.78
Diarrhoea	35	1.83 (1.23 - 2.43)	1.61	2.04	0.594	2.58
Pyotraumatic dermatitis	34	1.78 (1.19 - 2.37)	1.40	2.15	0.287	2.57
Laceration	34	1.78 (1.19 - 2.37)	1.83	1.74	0.999	4.56
Alopecia	30	1.57 (1.01 - 2.12)	1.40	1.74	0.679	2.13
Gastroenteritis	29	1.52 (0.97 - 2.06)	1.40	1.63	0.812	3.57
Pododermatitis	28	1.46 (0.93 - 2.00)	0.86	2.04	0.050	2.42
Dermatitis	26	1.36 (0.84 - 1.88)	1.40	1.33	0.999	4.29
Undesirable behaviour	25	1.31 (0.80 - 1.82)	1.18	1.43	0.782	3.25
Overgrown nails	25	1.31 (0.80 - 1.82)	1.83	0.82	0.082	5.08
Demodicosis	25	1.31 (0.80 - 1.82)	1.72	0.92	0.182	1.29
Allergy hypersensitivity disorder	24	1.25 (0.76 - 1.75)	1.29	1.23	0.999	2.72
Ocular discharge	23	1.20 (0.71 - 1.69)	1.40	1.02	0.589	1.65
Periodontal disease	23	1.20 (0.71 - 1.69)	1.07	1.33	0.765	6.81
Umbilical hernia	22	1.15 (0.67 - 1.63)	1.18	1.12	0.999	1.02
Vomiting	22	1.15 (0.67 - 1.63)	1.61	0.72	0.105	2.26
Traumatic claw injury	19	0.99 (0.55 - 1.44)	0.75	1.23	0.417	2.63

Of the 51 group-level disorder terms reported during 2013, the most common were ophthalmologic (*n =* 426*,* prevalence = 22.27%, 95% CI: 20.40-24.13), dermatological (*n =* 402*,* 21.01%, 95% CI: 19.19-22.84), aural (*n =* 357*,*  18.66%, 95% CI: 16.92-20.41), traumatic injury (*n =* 144*,*  7.53%, 95% CI: 6.35-8.71), and behavioural (*n =* 138*,*  7.21%, 95% CI: 6.05-8.37) (Table 3). Among the 20 most common group-level disorders, males had higher probability of behavioural disorders (*P* <  0.001, two-proportion *z*-test). The median age of dogs with the most common group-level disorders ranged from 1.05 years for hernia to 8.00 years for neoplasia (Table 3).

**Table 3:** Prevalence of the most common disorders at group-level diagnostic precision in Shar Pei (*n* = 1913) under primary veterinary care at practices participating in the VetCompass™ Programme in the UK from January 1st to December 31st, 2013. * *P*-value for prevalence comparison between male and female with two-proportion *z*-test

Group-level disorder	Count	Prevalence (%)	Female prevalence (%)	Male prevalence (%)	*P*-value*	Median age (years) of dogs with the condition
Ophthalmologic	426	22.27 (20.40 - 24.13)	23.42	21.14	0.255	1.53
Dermatological	402	21.01 (19.19 - 22.84)	20.52	21.45	0.656	2.61
Aural	357	18.66 (16.92 - 20.41)	19.44	17.88	0.412	2.80
Traumatic injury	144	7.53 (6.35 - 8.71)	7.95	7.15	0.566	3.10
Behavioural	138	7.21 (6.05 - 8.37)	4.08	10.21	< 0.001	3.36
Enteropathy	123	6.43 (5.33 - 7.53)	5.48	7.35	0.115	2.89
Mass associated	76	3.97 (3.10 - 4.85)	3.76	4.19	0.717	4.96
Musculoskeletal	72	3.76 (2.91 - 4.62)	4.08	3.47	0.563	3.44
Shar Pei Fever	58	3.03 (2.26 - 3.80)	2.90	3.17	0.837	4.45
Claw disorder	48	2.51 (1.81 - 3.21)	2.90	2.15	0.364	3.54
Parasitic	48	2.51 (1.81 - 3.21)	3.11	1.94	0.136	1.29
Upper respiratory tract	37	1.93 (1.32 - 2.55)	1.93	1.94	0.999	1.30
Lethargy	36	1.88 (1.27 - 2.49)	1.93	1.84	0.999	3.08
Female reproductive (females only)	35	1.83 (1.23 - 2.43)	3.76	–	–	2.11
Neoplasia	33	1.73 (1.14 - 2.31)	2.04	1.43	0.396	8.00
Dental	31	1.62 (1.05 - 2.19)	1.61	1.63	0.999	6.61
Hernia	30	1.57 (1.01 - 2.12)	1.40	1.74	0.679	1.05
Complication associated with clinical care	19	0.99 (0.55 - 1.44)	1.29	0.72	0.302	2.18
Male reproductive (males only)	17	0.89 (0.47 - 1.31)	0-	1.74	–	2.16
Urinary	16	0.84 (0.43 – 1.24)	1.18	0.51	0.175	3.44

**Fig. 3:**
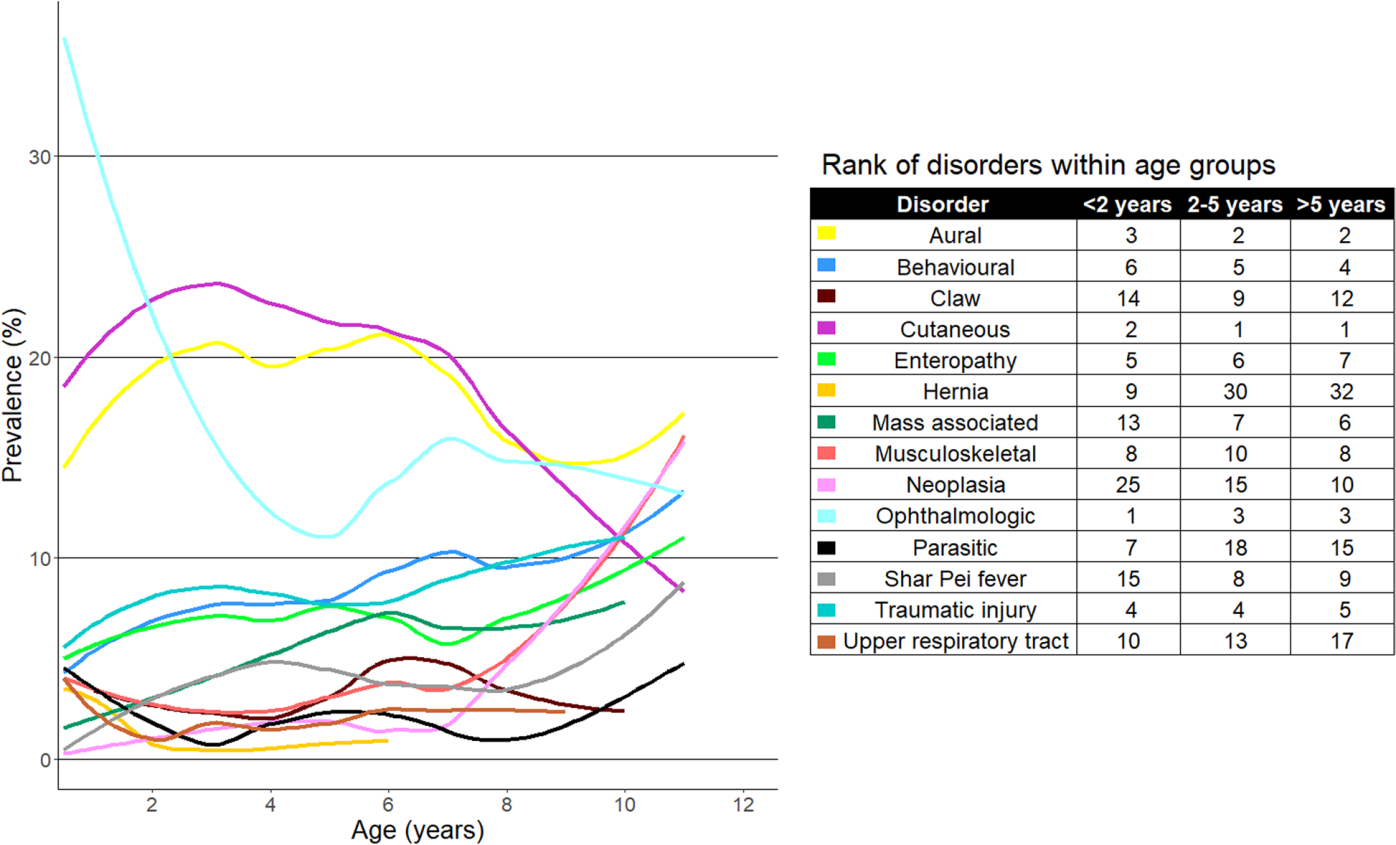
Prevalence of the 10 most common group-level disorders within each of three age bands (under 2 years *n = 718*, 2–5 years *n = 681*, over 5 years *n = 493*) in Shar Pei dogs under primary veterinary care at UK practices participating in the VetCompass™ Programme from January 1st to December 31st, 2013

The prevalence of the top 10 most common group-level disorders in three age bands: < 2 years (*n* = 718), 2 - 5 years (*n* = 681), and > 5 years (*n* = 493) is presented in Fig. 3. The prevalence of behavioural disorders, mass associated disorders, hernias, ophthalmological disorders, parasitic infections, Shar Pei fever and neoplasia (7/14 disorders, 50.00%) varied significantly between the age groups (chi-square test, *P* <  0.05).

### Mortality

Overall, 190/1913 (9.93%) Shar Pei had evidence of death during the study period. The median age at death was 7.28 years (IQR 5.04-10.05, range 0.04-15.04). The longevity of females (median 8.01 years, IQR 5.62–10.10, range 1.20-15.04, *n* = 94) did not differ statistically to males (median 6.88 years, IQR 4.39–9.95, range 0.04–14.60, *n* = 96) (Mann-Whitney *U* test, *P* = 0.077). Of 184 (96.84%) deaths with a recorded method of death, 157 (85.33%) deaths involved euthanasia and 27 (14.67%) deaths were unassisted. Among 136 (71.58%) deaths with a recorded biomedical cause of death, the most common causes of death at group level precision were neoplasia (*n =* 21, 15.44%, 95% CI: 9.37-21.51), renal disorders (*n =* 18, 13.24%, 95% CI: 7.54-18.93), and behavioural disorders (*n =* 15, 11.03%, 95% CI: 5.76-16.29) (Table 4).

**Table 4:** Mortality in Shar Pei dogs (*n* = 136) with a recorded cause of death under primary veterinary care at UK practices participating in the VetCompass™ Programme from January 1st to December 31st, 2013. ^a^*CI* confidence interval

Group-level disorder	Count	Percent (95% CI^a^)
Neoplasia	21	15.44 (9.37 - 21.51)
Renal disorder	18	13.24 (7.54 - 18.93)
Behavioural disorder	15	11.03 (5.76 - 16.29)
Mass-associated disorder	12	8.82 (4.06 - 13.59)
Enteropathy	11	8.09 (3.51 - 12.67)
Hepatopathy	7	5.15 (2.52 - 10.24)
Brain disorder	6	4.41 (2.04 - 9.29)
Heart disorder	5	3.68 (1.58 - 8.32)
Poor quality of life	5	3.68 (1.58 - 8.32)
Dermatological disorder	5	3.68 (1.58 - 8.32)
Collapsed	3	2.21 (0.75 - 6.28)
Immune-mediated disorder	3	2.21 (0.75 - 6.28)
Lower respiratory tract disorder	3	2.21 (0.75 - 6.28)
Upper respiratory tract disorder	3	2.21 (0.75 - 6.28)
Other	19	13.97 (8.14 - 19.80)

## Discussion

This study provides detailed information on the demography and disorder profiles of 1913 Shar Pei dogs under primary veterinary care during 2013 in the UK. Shar Pei comprised 0.42% of all UK dogs in the study. Assuming a UK dog population of 10 million dogs, this suggests that there were around 42,000 Shar Pei dogs in the UK at that time and therefore predisposition for disorders with high welfare impact could affect substantial numbers of dogs [31, 40, 41]. Although the current data are from 2013, 10-year registration statistics from the Kennel Club indicate minimal fluctuations in Shar Pei breed popularity in the intervening decade [8]. Filling the data gap on the most common disorders of Shar Pei, especially for those disorders linked to extreme conformations, could contribute to wider ongoing efforts to reform breed health towards conformations that protect rather than predispose dogs to health and welfare issues that are largely preventable [11, 21, 25]. Veterinary surgeons have long advised against breeding the current extreme forms of the Shar Pei, with a survey of New Zealand veterinary surgeons placing the Shar Pei second only to the English Bulldog as having health and welfare too compromised to continue breeding [42]. Furthermore, in that same study, the Shar Pei was the second most common breed (also after the English Bulldog) that veterinary surgeons advised clients against purchasing, specifically due to common problems with skin, entropion and aggression [42]. The current results can support the veterinary profession to provide better evidence-based advice to owners around purchasing a Shar Pei based on predictable health, welfare, financial, legal and ethical outcomes.

Ophthalmological conditions represented the most prevalent disorder group in the Shar Pei in the current study, with 22.27% of dogs affected. This prevalence is much higher than the 7.02% prevalence of ophthalmological disorders in dogs overall in the UK reported using a similar methodology, where ophthalmological disorders represented only the eight most common disorder group [33]. This comparison strongly suggests that Shar Pei are highly predisposed to ophthalmological conditions. At the level of precise disorder diagnoses, entropion was the single most common disorder diagnosed in Shar Pei, with 17.88% of the dogs diagnosed with entropion in that single year, suggesting entropion as the dominant driver for the frequent ophthalmological problems in the breed [43]. However, even the high prevalence reported in the current study may substantially underestimate the true scale of entropion in the Shar Pei, with The Blue Book from the American College of Veterinary Ophthalmologists reporting entropion in 50.7% of Shar Pei from 1991 to 2015 and 28.7% from 2016 to 2020 that were actively assessed for the condition [24]. Entropion carries high welfare costs for affected dogs, with constant rubbing of periorbital hairs or eyelashes causing direct pain and irritation to the sensitive corneal surface and leading to consequential ocular disorders such as corneal ulceration [44]. The extensive frontal skin folds and abundance of subcutaneous tissue on the head are considered the main factor underlying high rates of entropion in the Shar Pei, with blepharospasm following prolonged corneal irritation exacerbating the degree of entropion over time [18]. In efforts to encourage moving the Shar Pei away from the extreme conformation of skin folding with subsequent entropion that is so pervasive in the breed, The Kennel Club’s Breed Watch system specifically cites excessive loose facial skin with conformational defects of the upper and/or lower eyelids so that the eyelid margins are not in normal contact with the eye as a point of concern for judges to monitor [7]. The Kennel Club breed standard for the Shar Pei also explicitly requires that dogs are ‘free from entropion’ [16]. However, only Breed Watch Category 3 breeds (currently 15 breeds) are mandated to undergo a veterinary health check at major Kennel Club dogs shows, and therefore the Shar Pei as a Category 2 breed does not receive this veterinary health check at shows [45]. There is currently increasing focus on producing dogs with good innate health and and on avoiding normalisation of extremes of conformation that are so common but avoidable in many breeds [46]. Endemically high rates in the Shar Pei highlight the entropion anatomic condition as a welfare priority for immediate breeding reform, especially in the face of evidence that modern dog producers prioritise breeding ‘meat mouth’ Shar Pei with extreme conformation over moving back to more moderate ‘bone mouth’ conformations that may carry less health costs to the dogs but may make dogs less saleable or less prize-worthy in the show ring [47]. In addition to discouraging the general public from choosing to acquire dogs with extreme conformations, by raising awareness to owners of the high prevalence of entropion in Shar Pei, veterinary professionals can also partner more effectively with owners to ensure prompt veterinary care for dogs already suffering the effects of entropion [48]. It is also critical that dog producers, the dog showing community and buyers do not normalise routine conformation-altering surgeries such as ‘eye tacking’ of young Shar Pei puppies with entropion as an ethically acceptable option to justify ongoing breeding of dogs with extreme conformation [49].

Dermatological disorders were the second most prevalent disorder group overall in Shar Pei and were recorded in 21.01% of dogs. This value is substantially higher than the 12.58% prevalence of dermatological disorders recorded in dogs overall using a similar methodology [33]. Aural disorders were the third most common disorder group in Shar Pei in the current study and showed a prevalence of 18.66% that was also substantially higher than the 8.17% prevalence reported for aural disorders in dogs overall in the UK [33]. These results suggest that the Shar Pei is strongly predisposed to skin and aural disorders and that the underlying risk factors for these two conditions may be linked. At a fine level of diagnostic precision in the current study, otitis externa with a prevalence of 16.36% represented the second most common diagnosis in Shar Pei. This value is much higher than the 7.3% prevalence reported across all dogs in the UK study of otitis externa using a similar methodology and where predisposition in the Shar Pei was identified with 3.44 times the odds of otitis externa compared to crossbred dogs [20]. Although otitis externa is recognised as a multifactorial disorder with several primary, secondary, predisposing and perpetuating factors potentially triggering each clinical episode, extreme conformational factors from thickened folded skin and subcutaneous tissues leading to stenotic ear canals are likely to strongly predispose the Shar Pei to otitis externa [19, 50]. Similarly to the breed-based reforms discussed above to reduce the prevalence of entropion, moves away from extremes of conformation related to skin folding and thickened skin could also promote greater aural ventilation and skin health in the ear canal.

Aggression with a prevalence of 5.23% was the fourth highest fine-level disorder in Shar Pei in the current study. This high probability of aggression in Shar Pei is more than double the 2.24% recorded in dogs overall using a similar methodology where aggression was only the 10th most commonly recorded disorder of dogs. Concerns about high aggression in Shar Pei have been raised for many years, with a survey of veterinarians in New Zealand in 1996 reporting the Shar Pei breed as the seventh highest for aggression from 132 breeds assessed [51]. High aggression might perhaps be expected historically for a breed originally developed as a fighting and hunting dog [1]. However, given that the majority of Shar Pei in the UK are now more likely to be owned as a domestic companion rather than for fighting, a stable, biddable and less aggressive temperament becomes a key criterion when deciding on what type of family dog to acquire [52]. Among the Shar Pei dogs in the current study, the probability of aggression varied hugely between males (7.97% affected) and females (2.36% affected). This predisposition for aggression in male dogs in Shar Pei concurs with a wide literature reporting higher levels of aggression in male dogs [33, 53–56]. Although expression of aggression in dogs can follow a range of assumed motivating factors such as fear, pain or territoriality, in many cases the true motivations for any specific aggressive event remain unknown [56]. However, awareness of a general predisposition towards aggression in Shar Pei may assist owners in their decision-making when considering which dog breed to introduce into their family home. For owners still committed to acquiring a Shar Pei, the current evidence suggests that choosing a female animal may offer prospects of reduced aggression compared to acquiring a male dog. However, aggression is a complex behaviour with genetic, hormonal and environmental influences. Awareness of the role of chronic pain as a contributing factor to aggressive behaviour in dogs has increased in recent years, with pain considered to reduce the threshold for the expression of aggression. Given the propensity towards multiple pain-inducing disorders in the Shar Pei, it is possible that moving the Shar Pei away from its current extreme conformation that promotes pain from conditions such as ocular irritation, skin fold dermatitis or otitis externa could also potentially reduce expression of aggression [57, 58]. Canine aggression is increasingly recognised as a serious concern for both for public and family safety as well as for the welfare of dogs themselves, so reducing the propensity to aggression in dogs kept for companion purposes should become a higher priority [59].

The existence of a disorder that is eponymous with one breed is suggestive of a critical genetic issue with that breed that, depending on the prevalence, duration and severity, may need urgent address to protect the welfare of dogs of that breed [31]. Shar Pei fever (also known as familial Shar Pei fever, periodic Shar Pei fever, Shar Pei recurrent fever syndrome, swollen hock syndrome) is a good example of a breed-limited disorder that merits serious consideration from legal, ethical, moral and welfare perspectives [48]. A genetic basis for Shar Pei fever has been identified that is linked to mankind’s strong selection for the distinctive thickened and folded skin that occurred early in popularisation of the Shar Pei breed as a domestic pet [13, 26, 27]. From a prevalence perspective, the current study reports Shar Pei fever as the ninth most commonly diagnosed disorder, with 3.03% of Shar Pei dogs diagnosed with the condition each year. However, even this frequency may severely under-estimate the true scale of the issue. A survey of owners of Shar Pei in the UK reported that only around half of events of Shar Pei fever in their dogs resulted in active veterinary care. For dogs affected with Shar Pei fever, the clinical picture is often severe, with fevers that can reach 107^o^F typically lasting 12 to 36 hours and linked with listlessness, loss of appetite and often swelling of the hocks. There may also be associated diarrhoea and the muzzle may swell and become painful [60]. As well as being directly unpleasant, the fever episodes can be accompanied by development of renal amyloidosis that destroys the kidney’s ability to filter proteins with the proteinuria leading to a propensity for abnormal blood clots throughout the body and high blood pressure [60]. Although intermittent, the fever episodes tend to be highly recurrent, with a median of four episodes per year reported in affected dogs from an owner survey in the UK [5]. Taken together, the high prevalence, severity and duration of Shar Pei fever suggest that this condition should be considered as a major welfare priority for this breed, especially given that the condition is largely limited to this one breed and has been exacerbated by mankind’s inexorable push towards selecting for more and more extreme conformations in the dogs that we prefer to own [5, 11, 31].

Promoting the ability of dogs to experience a good quality of life is enshrined in animal welfare legislation for England and Wales [61], Scotland [62] and Northern Ireland [63]. These various forms of legislation such as Section 9 of the Animal Welfare Act 2006 require that the welfare needs of animals must be met including the need to be protected from pain, suffering, injury and disease. From a legislative perspective, this places every dog-owner under a legal obligation to ensure adequate levels of medical and husbandry care to reasonably prevent pain, suffering, injury and disease in their dog. Meeting this duty is likely to be much more onerous for owners who choose to own dogs with extreme conformations because of the inherently higher health challenges in such dogs. Anyone considering acquiring a dog with an extreme conformation should bear in mind the responsibility of these legal requirements before making a final decision on which type of dog to acquire. Specifically in relation to anyone considering breeding dogs in England, Sch. 6, 6(5) Animal Welfare (Licensing of Activities Involving Animals) (England) Regulations 2018 [64] states that ‘*No dog may be kept for breeding if it can reasonably be expected, on the basis of its genotype, phenotype or state of health that breeding from it could have a detrimental effect on its health or welfare or that of its offspring*’. Similar legislation is also in place in Scotland in Sch. 6, 8(5) Animal Welfare (Licensing of Activities Involving Animals) (Scotland) Regulations 2021) [65]. Considering this legislation specifically in relation to the Shar Pei, given the strength of evidence that a genotype with a genetic mutation in the HAS2 gene predictably leads to hyaluronsosis with many detrimental effects on the health and welfare of the offspring [12, 13], it is reasonable to conclude that breeding from dogs with the HAS2 gene mutation breaches the current legislation in relation to licenced breeders. Wider public awareness of their legal obligations in relation to extreme conformations for anyone considering acquiring, owning or breeding a dog could help to dampen the current human fascination for promoting extreme body shapes in dogs that do not exist in nature. There is currently work underway to develop greater clarity on the intent, interpretation and implications of current legislation across the various regions of the UK in relation to extremes of conformation in dogs [10].

The current study had some limitations. The study applied a secondary use as a research resource to clinical data from primary-care veterinary practices. Although this offered benefits from reflecting all disorders recorded in  the study dogs and that these diagnoses were made to primary care veterinary standards, it is also possible that the results underestimated conditions that were less likely to lead to veterinary presentation or to being formally recorded in the clinical records [66]. Normalisation of disorders within breed that are de facto an extreme conformation (e.g. entropion) or are closely linked to an extreme conformation (e.g. skin fold dermatitis) may mean that these disorders are considered by owners and veterinarians as ‘normal for breed’ and thus may be under-reported in veterinary clinical data [67]. Many conditions in veterinary primary care are recorded using the main presenting sign (e.g. diarrhoea) rather than the formal biomedical terms (e.g. enteritis) because full diagnostic work-up is not always necessary or even appropriate in primary care practice where resolution of the clinical issue is often the main aim rather than a focus on elucidating a precise name for the condition [68]. The mortality analysis included relatively few dogs so the results should be considered with caution. The study included many individual analyses so there is a higher possibility of some Type I error (false positives) [69]. Consequently, the current results for individual comparisons should be considered as exploratory rather than confirmatory and wider inference should also consider the breadth of previous literature as well. The study relied on data from dogs under veterinary care in 2013 and diagnostic profiles of the breed may have changed in the intervening time. Although the Royal College of Veterinary Surgeons (RCVS) does maintain a register of veterinary surgeons, there is no complete national register of veterinary clinics in the UK. The RCVS does maintain a register of veterinary practice premises accredited by the RCVS Practice Standards Scheme that included 3235 premises in 2020 but the true number of clinics is likely to be substantially higer [70]. The current study sample of 304 clinics therefore represents under 10% of all UK clinics and therefore there is a possibility of selection bias for the clinic, veterinary professional, owner and dog demographics included in the current analysis. Since the work done on the current study, VetCompass has continued to accrue more practices to participate in this national welfare-focused research programme and currently has over 1800 UK participating clinics [32]. Consequently, newer studies could mitigate any selection biases within the current work.

## Conclusions

This study provides prevalence data for common disorders in the Shar Pei breed and specifically highlights that almost one fifth of the breed receive veterinary care each year for entropion. This painful extreme conformation is strongly linked to the hereditary disease of hyaluronosis which largely defines the breed with characteristic thickened and folded skin. Several other common disorders in the current study are also linked to hyaluronosis. The findings from the current study can be used by veterinary professionals as an evidence base to encourage clients considering acquiring a dog to prioritise the welfare of the dog over any human desires to own a dog with an extreme conformation, in line with shared national messaging from UK stakeholders in canine welfare [11, 21]. For current owners of Shar Pei dogs, the findings here provide strong evidence on the key health concerns that are genetically determined in the breed and that require high levels of owner and veterinary care to prevent long-term suffering, and also explains that while these disorders may be common in the breed, they are not normal for dogs. For legislators, the findings here provide a strong evidence base to help assess whether the dogs that we currently define as Shar Pei meet the requirements of The Animal Welfare Act (2006) that includes the need to be protected from pain, suffering, injury and disease, and The Animal Welfare (Licensing of Activities Involving Animals) Regulations 2018 that states that ‘No dog may be kept for breeding if it can reasonably be expected, on the basis of its genotype, phenotype or state of health that breeding from it could have a detrimental effect on its health or welfare or that of its offspring’ [48, 61, 64]. In summary, it is clear that several serious welfare issues are genetically and phenotypically intrinsic to the current extreme variants of Shar Pei that are commonly owned in the UK and that urgent action is needed to reform the breed to protect the welfare of dogs and to ensure that anyone producing, owning or promoting these dogs in the future is meeting their legal, ethical, moral and social responsibilities.

## Data Availability

The datasets generated during and/or analysed during the current study will be made available at the RVC Research Online repository.

## Abbreviations

CI: confidence interval
EPR: electronic patient record
IQR: interquartile range
KC: The Kennel Club
RVC: Royal Veterinary College
SD: standard deviation
